# Prävalenz kraniomandibulärer Dysfunktionen bei Senioren – symptombezogene Analysen bei jüngeren und älteren Senioren

**DOI:** 10.1007/s00391-021-01954-z

**Published:** 2021-08-05

**Authors:** Angelika Rauch, Cäcilie Angrik, Andreas Zenthöfer, Sophia Weber, Sebastian Hahnel, Ina Nitschke, Oliver Schierz

**Affiliations:** 1 Poliklinik für Zahnärztliche Prothetik und Werkstoffkunde, Universität Leipzig, Liebigstr. 12, Haus 1, 04103 Leipzig, Deutschland; 2 Gemeinschaftspraxis Koch, Wichernstr. 1, 01445 Radebeul, Deutschland; 3 Poliklinik für Zahnärztliche Prothetik, Universitätsklinikum Heidelberg, Im Neuenheimer Feld 400, 69120 Heidelberg, Deutschland; 4 Klinik für Allgemein‑, Behinderten- und Seniorenzahnmedizin, Zentrum für Zahnmedizin, Universität Zürich, Plattenstr. 11, 8032 Zürich, Schweiz

**Keywords:** Kiefergelenk, Kiefergelenkknacken, Orofaziale Schmerzen, Chronische Schmerzen, Ältere, Temporomandibular Joint, Clicking, Orofacial pain, Chronic pain, Aged

## Abstract

**Hintergrund:**

Kraniomandibuläre Dysfunktionen (CMD) können auch im hohen Alter auftreten. Die Prävalenz von CMD-Symptomen bei Senioren wurde bisher nur wenig untersucht.

**Ziel der Arbeit:**

Ziel dieser Untersuchung war es, die Prävalenz von Symptomen einer CMD bei Senioren nach Befunderhebung mit den Research Diagnostic Criteria for Temporomandibular Disorders (RDC/TMD) zu bestimmen. Dabei sollten die Prävalenzwerte von jüngeren (60 bis 74 Jahren) und älteren (≥ 75 Jahre) Senioren verglichen werden.

**Material und Methoden:**

Im Rahmen der Interdisziplinären Längsschnittstudie des Erwachsenenalters (ILSE) wurden Probanden nach repräsentativen Gesichtspunkten rekrutiert. Während der vierten Nachverfolgungswelle im Zeitraum von 2014 bis 2016 im Bereich des Studienzentrums Leipzig wurden die Probanden auf das Vorliegen von anamnestischen und klinischen CMD-Symptomen untersucht.

**Ergebnisse:**

Anamnestische CMD-Symptome bei Senioren (*n* = 192) waren v. a. durch Schmerzen im Gesichtsbereich (13,0 %) gekennzeichnet. Das häufigste klinische CMD-Symptom waren Kiefergelenkgeräusche mit einer Prävalenz bis zu 35,5 %. Frauen gaben anamnestisch häufiger Kopfschmerzen/Migräne an. Kiefergelenkgeräusche und eine limitierte Mundöffnung wurden klinisch häufiger bei weiblichen Teilnehmenden beobachtet. Statistisch signifikante Unterschiede zeigten sich bei dem Vergleich von jüngeren und älteren Senioren hinsichtlich der Prävalenz von Kopfschmerzen/Migräne, jedoch nicht bei klinischen Symptomen.

**Schlussfolgerung:**

Anamnestisch werden Gesichtsschmerzen von 13,0 % der Senioren angegeben. Kiefergelenkgeräusche werden bei jedem dritten Älteren klinisch beobachtet. CMD-Symptome scheinen bei jüngeren und älteren Senioren im ähnlichen Maße ausgeprägt zu sein.

## Abstract (en)

**Background:**

Temporomandibular disorders (TMD) are common in the general population even in old age. Information on the prevalence of TMD in older people is sparse.

**Objective:**

To present prevalence values for symptoms and signs of TMD in old people according to the research diagnostic criteria for temporomandibular disorders (RDC/TMD). To compare prevalence values between the age groups of 60–74 years (younger seniors) and ≥ 75 years (older seniors).

**Material and methods:**

Subjects of the interdisciplinary longitudinal study of adult development (ILSE), a representative sample of old people in Germany, were examined between 2014–2016. Subjects of the urban area of Leipzig were chosen to examine symptoms and signs of TMD.

**Results:**

The most prevalent symptom was orofacial pain (13.0%) in the aged participants (*n* = 192). Temporomandibular joint (TMJ) sounds were the most prevalent sign with values up to 35.5%. Gender comparisons revealed higher prevalence values for the symptom headache/migraine as well as for the signs TMJ sounds (especially clicking sounds) and limited mouth opening in females. Regarding age groups, significant differences were only observed for self-reported headache/migraine.

**Conclusion:**

Orofacial pain was reported by 13.0% of the subjects and TMJ sounds were observed in every third participant. Similar prevalence values for TMD symptoms and signs in younger and older seniors were found.

## Vorspann

Kraniomandibuläre Dysfunktionen (CMD), welche durch Schmerzen im Gesichtsbereich, Funktionseinschränkungen der Unterkieferbeweglichkeit oder Kiefergelenkgeräusche gekennzeichnet sind, können auch im hohen Alter auftreten. Die muskulären und/oder arthrogenen Beschwerden können durch verschiedene Faktoren wie z. B. psychosoziale Charakteristika, allgemeine Erkrankungen oder Zähneknirschen beeinflusst werden. Über die Häufigkeit des Auftretens von CMD-Symptomen bei Senioren ist bisher wenig bekannt. Im Rahmen der interdisziplinären Längsschnittstudie im Erwachsenenalters (ILSE) wurden Prävalenzwerte für CMD-Symptome bei jüngeren und älteren Senioren erfasst, welche im Nachfolgenden vorgestellt werden.

## Hintergrund und Fragestellung

Schmerzen im Gesichtsbereich, Limitationen der Kieferbeweglichkeit oder Kiefergelenkgeräusche können in einer erheblichen Beeinträchtigung der mundgesundheitsbezogenen Lebensqualität der Betroffenen resultieren [13]. Diese genannten Beschwerdebilder sind die Kardinalsymptome der CMD – ein Erkrankungsbild, dass die häufigste Ursache für nonodontogene Schmerzen im orofazialen Gebiet darstellt [15]. Die Inzidenz für das Auftreten einer CMD liegt bei 4 % pro Jahr [30]. Für die Gesamtbevölkerung werden Prävalenzwerte im Bereich von 5 bis 12 % angegeben [24]. In der Vergangenheit wurde jedoch deutlich, dass alters- und geschlechtsspezifische Untersuchungen notwendig sind, um die Ätiopathogenese und den Verlauf der CMD besser verstehen zu können. Es zeigte sich, dass Frauen über alle Altersgruppen hinweg eine höhere Anfälligkeit für CMD aufweisen. Das Verhältnis liegt je nach Studie zwischen 4:1 und 2:1 (Frauen:Männer) [15, 31].

Die Gründe für das Entstehen einer CMD sind bisher nicht vollständig erforscht; allerdings ist bekannt, dass psychosoziale Charakteristika und auch hormonelle Faktoren die Entstehung und Prognose einer CMD relevant beeinflussen können [7]. Bei Frauen nimmt die Häufigkeit von CMD-Symptomen im jugendlichen Alter mit Fortschreiten der weiblichen Pubertät zu [22]. Frauen im gebärfähigen Alter sind besonders häufig betroffen. Im Seniorenalter nehmen die CMD-assoziierten Symptome jedoch eher wieder ab [15, 29, 31]. Daten zur Prävalenz von CMD bei alten und sehr alten Personen, welche in einer standardisierten Untersuchung erhoben wurden, sind selten. Jedoch wird eine Verschiebung der Hauptbeschwerden im Seniorenalter von muskulär-assoziierten zu arthrogenen Symptomen beschrieben. Zugleich wurden schwer ausgeprägte CMD bei Senioren weniger häufig beobachtet [10, 28]. Die geschlechtsspezifischen Unterschiede in der Ausprägung von CMD-Symptomen scheinen im Seniorenalter wieder abzunehmen [3, 10].

Die Prävalenzwerte von CMD-Symptomen werden in der Literatur sehr heterogen beschrieben. Dies ist u. a. durch das Vorhandensein von mannigfaltigen Befunderhebungs- und Diagnosesystemen bedingt. Validierte und standardisierte Untersuchungskriterien nach internationalem Standard sollten daher zur Vergleichbarkeit mit anderen Studien herangezogen werden [33]. Die *Research Diagnostic Criteria for Temporomandibular Disorders* (RDC/TMD) erfüllen diese Anforderungen und sind zur klinischen Untersuchung von CMD international etabliert [6, 16]. Sie sind kostenfrei und in verschiedenen Sprachen, so auch in Deutsch, verfügbar [11].

Im Rahmen der interdisziplinären longitudinalen Studie des Erwachsenenalters (ILSE) sollten zwei Kohorten von jüngeren (60 bis 74 Jahre) und älteren (≥ 75 Jahre) Senioren untersucht werden. Ziel dieser Untersuchung war es, die Prävalenz von Symptomen einer CMD bei Senioren nach Befunderhebung mit den RDC/TMD zu bestimmen. Die Arbeitshypothese war, dass es keine Unterschiede in den Häufigkeiten von anamnestischen und klinischen Symptomen zwischen den beiden Kohorten gibt.

## Studiendesign und Untersuchungsmethoden

### Rekrutierung (ILSE) und Untersuchung

Die interdisziplinäre longitudinalen Studie des Erwachsenenalters (ILSE) ist eine in den Jahren 1993–1996 erstmals durchgeführte Untersuchung mit 1390 Probanden aus Ost- (Untersuchungszentren Leipzig und Rostock) und Westdeutschland (Untersuchungszentren Heidelberg, Bonn und Erlangen-Nürnberg). Die selbstständig lebenden Probanden wurden stratifiziert nach Geschlecht, Wohnort und Kohortenzugehörigkeit (Jahrgänge 1930–1932 und 1950–1952) aus den Einwohnermelderegistern ausgewählt. Bei den Probanden wurden demografische Angaben und gesundheitsspezifische Parameter erhoben. Weiterführende Informationen zu der ILSE-Studie sind online und in zahlreichen Publikationen verfügbar [25, 27]. Im Rahmen der vierten Untersuchungswelle im Zeitraum von 2014 bis 2016 im Gebiet Leipzig wurden die Probanden auch auf das Vorliegen von CMD-Symptomen untersucht. Zur Erhebung von anamnestischen Symptomen wurde ein Selbstfragebogen genutzt, den die Probanden im Vorfeld der Untersuchung ausfüllen sollten. Folgende Fragen wurden gemäß der Vorlage des *Patient History Questionnaire* (PHiQ) der RDC/TMD formuliert und ausgewertet [12].

- Hatten Sie Schmerzen im Gesicht, im Kiefer, in den Schläfen, vor dem oder im Ohr im vergangenen Monat? (Entspricht PHiQ F3)
- War Ihr Unterkiefer jemals blockiert, oder hatten Sie Schwierigkeiten, den Mund vollständig zu öffnen? (Entspricht PHiQ F14a)
- Knackt es in Ihrem Kiefergelenk, wenn Sie den Mund öffnen oder schließen, oder wenn Sie kauen? (Entspricht PHiQ F15a)
- Hatten Sie während der vergangenen 6 Monate Probleme mit Kopfschmerzen oder Migräne? (Entspricht PHiQ F18)

Die klinische Befunderhebung nach RDC/TMD erfolgte durch eine in der Untersuchungstechnik trainierte Zahnärztin (C.A.). Alle Probanden gaben ihr schriftliches Einverständnis zur Teilnahme an der Studie. Die Studie wurde nach den Maßgaben der Deklaration von Helsinki durchgeführt und von der Ethikkommission der Medizinischen Fakultät an der Universität Leipzig genehmigt (Nr. 341/13-ff).

### Statistische Analyse

Die statistische Auswertung (SPSS 25, IBM, Armonk, New York, USA) umfasste die deskriptive Statistik und den Vergleich beider Kohorten mittels Chi-Quadrat-Tests. Die Kohorten setzten sich aus den um 1950 geborenen jüngeren (60 bis 74 Jahren) und den um 1930 geborenen älteren (≥ 75 Jahre) Senioren zusammen. Das Signifikanzniveau wurde bei *α* = 0,050 festgelegt. Die Strukturen der Mm. temporales und der Mm. masseteres wurden als Kaumuskulatur betrachtet. Die Befunde für Kiefergelenkgeräusche (Knacken oder Reiben) bei vertikalen, lateralen und protrusiven Unterkieferbewegungen wurden unabhängig von der Seite zusammengefasst. Die Latero- und Protrusionsbewegungen des Unterkiefers wurden als Exkursionsbewegungen angesehen. Eine limitierte Mundöffnung wurde bei einem Wert < 40 mm angenommen [26].

## Ergebnisse

Von April 2014 bis November 2016 wurden 192 Probanden nachuntersucht. Das durchschnittliche Alter lag bei 71,4 ± 9,4 Jahren (62 bis 86 Jahre). Von 191 Probanden waren 46,6 % weiblich; bei einem Probanden fehlte die Angabe des Geschlechtes. Die häufigsten anamnestischen CMD-Symptome waren Kopfschmerzen/Migräne (17,4 %) und Schmerzen im Gesichtsbereich (13,0 %). Während der klinischen Untersuchung wurden bei 32,0 % der Teilnehmenden ein Kiefergelenkgeräusch und bei 10,0 % eine schmerzfreie limitierte Mundöffnung festgestellt.

### Geschlechterspezifischer Vergleich

#### Anamnestische CMD-Symptome

**Figure 1:**
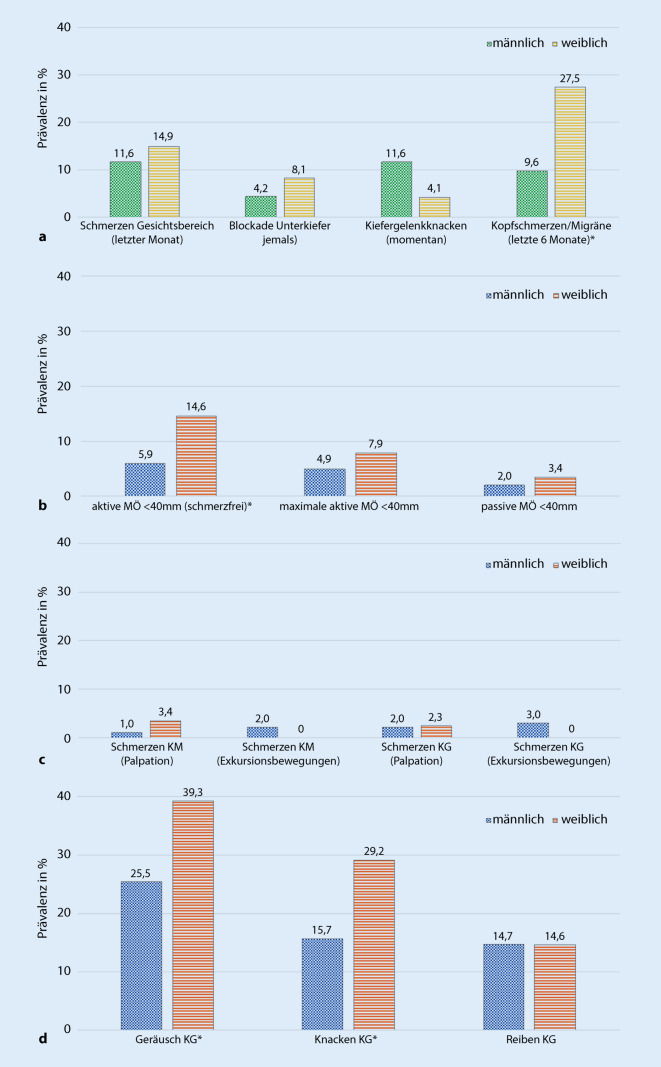

Die häufigsten anamnestischen Symptome waren Schmerzen im Gesichtsbereich. Ferner berichteten Frauen häufig über Kopfschmerzen/Migräne und Männer über ein Kiefergelenkknacken (Abb. 1a). Bei dem geschlechterabhängigen Vergleich der anamnestischen CMD-Symptome stellte sich für Kopfschmerzen/Migräne ein statistisch signifikanter Unterschied dar (*p* = 0,025).

#### Klinische CMD-Symptome

Die häufigsten klinischen Symptome waren Kiefergelenkgeräusche im Sinne eines Kiefergelenkknackens in beiden Geschlechtern (Abb. 1b, c). Die männlichen Probanden zeigten jedoch statistisch signifikant weniger Kiefergelenkgeräusche (i.e. Kiefergelenkknacken) als die weiblichen Teilnehmenden (*p* = 0,041 Geräusch; *p* = 0,024 Knacken). Dahingegen waren reibende Geräusche in den Kiefergelenken in beiden Geschlechtern mit ca. 15 % ähnlich häufig vertreten. Eine maximale aktive und schmerzfreie Mundöffnung unter 40 mm zeigte sich bei einem signifikant größeren Anteil der weiblichen als der männlichen Probanden (*p* = 0,044).

### Vergleich der jüngeren und älteren Senioren

#### Anamnestische CMD-Symptome

Geschlechtsunabhängig zeigten sich klinisch relevante Unterschiede zwischen den jüngeren und älteren Senioren bei den anamnestisch erhobenen Kiefergelenkgeräuschen (10,8 % vs. 3,5 %), welche jedoch nicht statistisch signifikant waren (Tab. 1). Eine signifikant größere Häufigkeit von Gesichtsschmerzen war bei den älteren weiblichen Senioren im Vergleich zu den jüngeren zu beobachten (*p* = 0,011).

**Table 1:** 

Anamnestische Symptome	Jüngere Senioren		Ältere Senioren		*p*-Wert
	64,4 ± 1,4 (62–74) Jahre		83,6 ± 1,2 (81–86) Jahre		
	*n*	Prävalenz in % (*n*)	*n*	Prävalenz in % (*n*)	
*Schmerzen Gesichtsbereich* *(letzter Monat)*	111	11,7 (13)	58	15,5 (9)	n. s.
Männlich	60	15,0 (9)	35	5,7 (2)	n. s.
Weiblich	51	7,8 (4)	23	30,4 (7)	0,011*
*Blockade Unterkiefer* *(jemals)*	111	5,4 (6)	58	5,2 (3)	n. s.
Männlich	60	3,3 (2)	35	2,9 (1)	n. s.
Weiblich	51	7,8 (4)	23	8,7 (2)	n. s.
*Kiefergelenkgeräusche* *(momentan)*	111	10,8 (12)	58	3,5 (2)	n. s.
Männlich	60	15,0 (9)	35	5,7 (2)	n. s.
Weiblich	51	5,9 (3)	23	0,0 (0)	n. s.
*Kopfschmerzen/Migräne* *(letzte 6 Monate)*	67	19,4 (13)	25	12,0 (3)	n. s.
Männlich	36	8,3 (3)	16	12,5 (2)	n. s.
Weiblich	31	32,3 (10)	9	11,1 (1)	n. s.

Signifikanter Unterschied (*Asterisk*), nicht signifikant (*n.* *s.*)

#### Klinische CMD-Symptome

Die häufigsten klinischen Symptome waren Kiefergelenkgeräusche mit einem Anteil von 35,5 % bei den jüngeren und 26,1 % bei den älteren Senioren (Tab. 2). Schmerzhafte Palpationsbefunde oder Exkursionsbewegungen waren nur selten zu beobachten. Die Mundöffnung war in ca. 10 % der Fälle limitiert. Zwischen den beiden Kohorten konnte, auch im geschlechterspezifischen Vergleich, kein statistisch signifikanter Unterschied festgestellt werden.

**Table 2:** 

Klinische Symptome	Jüngere Senioren		Ältere Senioren		
	64,4 ± 1,4 (62–74) Jahre		83,6 ± 1,2 (81–86) Jahre		*p*-Wert
	*n*	Prävalenz in % (*n*)	*n*	Prävalenz in % (*n*)	
*Aktive MÖ* *<* *40* *mm (schmerzfrei)*	121	8,3 (10)	70	12,9 (9)	n. s.
Männlich	64	3,1 (2)	38	10,5 (4)	n. s.
Weiblich	57	14,0 (8)	32	15,6 (5)	n. s.
*Maximale aktive MÖ* *<* *40* *mm*	121	5,0 (6)	70	8,6 (6)	n. s.
Männlich	64	3,1 (2)	38	7,9 (3)	n. s.
Weiblich	57	7,0 (4)	32	9,4 (3)	n. s.
*Passive MÖ* *<* *40* *mm*	121	1,7 (2)	70	4,3 (3)	n. s.
Männlich	64	0,0 (0)	38	5,3 (2)	n. s.
Weiblich	57	3,5 (2)	32	3,1 (1)	n. s.
*Schmerzen Kaumuskulatur – Palpation*	121	2,5 (3)	70	1,4 (1)	n. s.
Männlich	64	1,6 (1)	38	0,0 (0)	n. s.
Weiblich	57	3,5 (2)	32	3,1 (1)	n. s.
*Schmerzen, Kaumuskulatur – Exkursionsbewegung*	121	0,8 (1)	70	1,4 (1)	n. s.
Männlich	64	1,6 (1)	38	2,6 (1)	n. s.
Weiblich	57	0,0 (0)	32	0,0 (0)	n. s.
*Schmerzen, Kiefergelenk – Palpation*	121	1,7 (2)	70	2,9 (2)	n. s.
Männlich	64	1,6 (1)	38	2,6 (1)	n. s.
Weiblich	57	1,8 (1)	32	3,1 (1)	n. s.
*Schmerzen, Kiefergelenk – Exkursionsbewegung*	121	2,5 (3)	70	2,9 (2)	n. s.
Männlich	64	4,7 (3)	38	5,3 (2)	n. s.
Weiblich	57	0,0 (0)	32	0,0 (0)	n. s.
*Geräusch, Kiefergelenk*	121	35,5 (43)	69	26,1 (18)	n. s.
Männlich	64	28,1 (18)	37	21,6 (8)	n. s.
Weiblich	57	43,9 (25)	32	31,3 (10)	n. s.
*Knacken, Kiefergelenk*	121	21,5 (26)	69	23,2 (16)	n. s.
Männlich	64	14,1 (9)	37	18,9 (7)	n. s.
Weiblich	57	29,8 (17)	32	28,1 (9)	n. s.
*Reiben, Kiefergelenk*	121	17,4 (21)	69	10,1 (7)	n. s.
Männlich	64	18,8 (12)	37	8,1 (3)	n. s.
Weiblich	57	15,8 (9)	32	12,5 (4)	n. s.

Signifikanter Unterschied (*Asterisk*), nicht signifikant (*n.* *s.*), *MÖ* Mundöffnung

## Diskussion

Die Ergebnisse dieser Untersuchung zeigen, dass anamnestische CMD-Symptome bei Senioren v. a. durch Schmerzen im Gesichtsbereich gekennzeichnet sind. Weibliche Senioren, welche älter als 80 Jahre waren, gaben häufig Kopfschmerzen/Migräne an. Während der klinischen Untersuchung wurden Kiefergelenkgeräusche mit einer Prävalenz bis zu 35,5 % festgestellt. Geschlechtsspezifische Unterschiede wurden für die Angabe von anamnestischen Kopfschmerzen/Migräne sowie bei klinisch-beobachteten Kiefergelenkgeräuschen und schmerzfreier limitierter Mundöffnung festgestellt. Der Vergleich der anamnestischen CMD-Symptome bei jüngeren und älteren Senioren zeigte eine statistisch signifikant höhere Prävalenz von Kopfschmerzen/Migräne bei den älteren Frauen. Klinische CMD-Symptome waren zwischen den Kohorten nicht signifikant unterschiedlich, daher wurde die Nullhypothese teilweise verworfen.

Die Prävalenzwerte, welche in der vorliegenden Studie ermittelt wurden, ergänzen Ergebnisse aus anderen Studiengruppen. Bei deutschen Senioren konnte bisher eine geringere Schmerzbeeinträchtigung durch CMD als bei jüngeren Erwachsenen beobachtet werden [10, 28]. Im Rahmen der ILSE-Untersuchungen konnte ebenfalls eine geringere Häufigkeit schmerzhafter klinischer CMD-Symptome festgestellt werden. Nichtsdestotrotz war die Häufigkeit von anamnestischen Gesichtsschmerzen und teilweise auch von Kopfschmerzen/Migräne relativ hoch. Dies ist womöglich dadurch zu erklären, dass zahn- bzw. zahnfleischbezogene Schmerzen im Vorfeld der Frage nicht explizit ausgeschlossen wurden [8]. Die aktive vertikale Unterkieferbeweglichkeit war bei ca. 10 % der Probanden eingeschränkt. Ähnliche Werte wurden auch von vietnamesischen Senioren (65 bis 74 Jahre), welche gemäß den DC/TMD klinisch untersucht worden waren, berichtet. Die asiatische Seniorenkohorte präsentierte außerdem eine Häufigkeit von 50,4 % für Kiefergelenkreiben [19]. Dieser Wert liegt deutlich über dem Wert von 21 %, welchen Schmitter et al. [28] beobachteten, und über dem im Rahmen der ILSE ermittelten Wert von 17,4 % bei deutschen Senioren. Die Unterschiede sind womöglich durch anatomische oder auch genetische Faktoren der verschiedenen Bevölkerungsgruppen erklärbar [29]. Generell ist das Auftreten von Kiefergelenkgeräuschen im Alter jedoch wenig überraschend, da bekannt ist, dass ab dem 60. Lebensjahr gehäuft degenerative Veränderungen der Kiefergelenke auftreten [9, 34]. Das Kiefergelenkreiben ist dabei ein häufiges Zeichen für pathologische Veränderungen wie z. B. osteoarthrotische Veränderungen. Diese sollten frühzeitig erkannt werden, um einer Einschränkung der Kieferbeweglichkeit und dem Entstehen von Schmerzen vorzubeugen. Die beginnende Osteoarthrose kann konservativ therapiert werden. Zu diesem Zweck kann z. B. eine Äquilibrierungsschiene angefertigt werden, um die Kiefergelenke zu entlasten [17]. Die Wahrnehmung eines Kiefergelenkknackens ist ein häufiges CMD-Symptom in der allgemeinen Bevölkerung und resultiert meist aus einer anterioren Diskusverlagerung mit Reposition [33]. Ein schmerzfreies Kiefergelenkknacken bedarf in der Regel keiner Therapie [23]. Eine Studie konnte mithilfe von MRT-Untersuchungen aufzeigen, dass Osteoarthrose und Diskusverlagerungen bei Senioren oft gemeinsam auftreten und dass eine „odds ratio“ von 2,9 besteht [20]. Auch allgemeine und altersassoziierte Erkrankungen wie M. Parkinson, rheumatoide Arthritis und andere Gelenkerkrankungen (insbesondere der Kniegelenke) stellen Risikofaktoren für eine CMD dar [2, 4, 14].

Hinsichtlich der Unterschiede zwischen den jüngeren und älteren Senioren konnte anhand der Ergebnisse der ILSE-Studie – mit Ausnahme bei dem mit CMD-assoziierten Symptom Kopfschmerzen/Migräne – keine statistisch signifikanten Unterschiede festgestellt werden. Auch eine in der Literatur beschriebene Reduktion von anamnestischen Symptomen bei älteren Frauen mit zunehmendem Alter konnte nicht bestätigt werden [3, 32].

Eine der Limitationen der Studie ist in den kleinen Kohortengrößen zu sehen; besonders der Vergleich von Untergruppen sollte kritisch betrachtet werden. Da die Kohorten bereits in den 1990er-Jahren rekrutiert wurden und diese während der vierten Untersuchungswelle bereits in das (hohe) Seniorenalter eingetreten waren, konnten nur 192 der ehemals 502 Teilnehmer aus Leipzig zur Nachuntersuchung einbestellt werden. Aufgrund der kleinen Kohortengröße der vierten Untersuchungswelle der ILSE wurde von einer Stratifizierung der Daten entsprechend potenzieller Kofaktoren für CMD-assoziierte Symptome wie z. B. psychosoziale Einflüsse [5], Okklusion [18] oder auch Bruxismus [1, 21] abgesehen. Auf eine Verifizierung der Kiefergelenksymptome mittels MRT wurde aufgrund des erheblichen finanziellen und organisatorischen Mehraufwands und unter Berücksichtigung der verminderten Belastbarkeit der Senioren verzichtet. Nichtsdestotrotz ermöglichen die Ergebnisse der ILSE-Untersuchung erstmals einen vergleichenden Überblick über CMD-Symptome bei jüngeren und älteren Senioren in Deutschland unter Verwendung internationaler standardisierter Kriterien. Die Ergebnisse zeigen, dass Kiefergelenkveränderungen auch im hohen Alter ein häufiges CMD-assoziiertes Symptom darstellen. Aus diesem Grund sollte die CMD von behandelnden Zahn-(Ärzten) als potenzieller Risikofaktor für eine eingeschränkte mundgesundheitsbezogene Lebensqualität oder schmerzhafte Verläufe in Betracht gezogen werden.

## Fazit für die Praxis

Auf ältere Menschen über 60 Jahre trifft Folgendes zu:

- Gesichtsschmerzen werden anamnestisch von 13,0 % angegeben.
- Kiefergelenkgeräusche sind ein häufiges klinisches Symptom (32,0 %).
- Frauen sind häufiger von Symptomen der kraniomandibulären Dysfunktion (CMD) betroffen als Männer.
- Symptome der CMD scheinen bei jüngeren und älteren Senioren im ähnlichen Maße ausgeprägt zu sein.
- Klinische CMD-Symptome werden häufiger beobachtet als subjektive CMD-assoziierte Beschwerden.
- Die Notwendigkeit einer therapeutischen Intervention bei klinischen CMD-Symptomen sollte daher patientenindividuell abgewogen werden.
